# Advanced glycation end-products exacerbate myocardial ischemia/reperfusion injury by promoting mitochondrial oxidative damage and PANoptosis in diabetes mellitus

**DOI:** 10.1016/j.redox.2026.104228

**Published:** 2026-05-20

**Authors:** Ji'e Yang, Jian Zhang, Rong Huang, Ke Meng, Jiayu Liang, Jingyi Lin, Jingpu Wang, Yiweng Wang, Yang Gao, Yanan Qu, Shiyu Hu, Jiatian Cao, Hao Su, Feng Zhang, Junbo Ge

**Affiliations:** a Department of Cardiology, The First Affiliated Hospital of USTC, University of Science and Technology of China, Hefei, Anhui, 230000, PR China; b Division of Life Science and Medicine, University of Science and Technology of China, Hefei, Anhui, 230000, PR China; c Department of Cardiology, Zhongshan Hospital, Fudan University, Shanghai, 200032, PR China; d Shanghai Institute of Cardiovascular Disease, Shanghai, 200032, PR China

**Keywords:** Advanced glycation end-products, Mitochondrial oxidative damage, PANoptosis, Myocardial ischemia/reperfusion injury, Diabetes mellitus

## Abstract

Diabetes mellitus exacerbates myocardial ischemia/reperfusion injury (MI/RI), but the underlying mechanisms remain unclear. The present study identifies advanced glycation end-products (AGEs) as a key pathological mediator. Upon hypoxia/reoxygenation (H/R) stimulation, AGEs-primed cardiomyocytes exhibited drastic mitochondrial oxidative damage, characterized by elevated mitochondrial reactive oxygen species (mtROS), loss of mitochondrial membrane potential, depletion of ATP, and increased release of mitochondrial DNA and cytochrome *c*. Mechanistically, AGEs impaired the mitochondrial antioxidant defense via the RAGE-Nrf2-SOD2 axis. Furthermore, AGEs activated the AIM2-ZBP1 PANoptosome, triggering PANoptosis characterized by concurrent upregulation of cleaved caspase-3, GSDMD, and *p*-MLKL. Mitochondrial oxidative damage was established as the causal upstream event, as a mitochondria-targeted antioxidant or RAGE silencing attenuated PANoptosis, while mtROS inducer (antimycin A) directly activated PANoptosis. Therapeutically, combination treatment with pyridoxamine (an AGEs inhibitor) and empagliflozin (an SGLT2 inhibitor) in diabetic mice potently suppressed AGEs accumulation, mitigated mitochondrial damage and PANoptosis, and significantly improved cardiac recovery post-MI/R. Thus, targeting the AGEs-mitochondrial damage-PANoptosis axis via combined pyridoxamine and empagliflozin represents a promising strategy to alleviate diabetic MI/RI.

## Highlights

• Advanced glycation end-products (AGEs) accumulate in myocardium and exacerbate myocardial ischemia/reperfusion injury (MI/RI) in diabetic animal model.
• AGEs trigger PANoptosis in cardiomyocytes via activation of the AIM2-ZBP1 PANoptosome and upregulation of multiple cell death executors (cleaved caspase-3, GSDMD, *p*-MLKL).
• AGEs, via their receptor RAGE, suppress the master antioxidant transcription factor Nrf2, leading to downregulation of mitochondrial superoxide dismutase 2 (SOD2). This axis is the key to impaired mitochondrial antioxidant defense in diabetic cardiomyocytes.
• Targeting the AGEs-mitochondrial oxidative stress-PANoptosis axis represents a promising and clinically translatable strategy for diabetic cardiovascular complications.
• Combination therapy with pyridoxamine (AGE inhibitor) and empagliflozin (SGLT2 inhibitor) potently inhibits AGEs formation and attenuates MI/RI in diabetic mice.

## Graphical abstract

AGEs accumulate in cardiomyocytes under diabetic conditions, impairing mitochondrial antioxidant defense through the RAGE-Nrf2-SOD2 axis. Upon myocardial ischemia/reperfusion insult, diabetic cardiomyocytes exhibit heightened susceptibility to mitochondrial oxidative damage, which in turn triggers the assembly of the AIM2-ZBP1 PANoptosome and ultimately activates PANoptosis.

**Figure:**
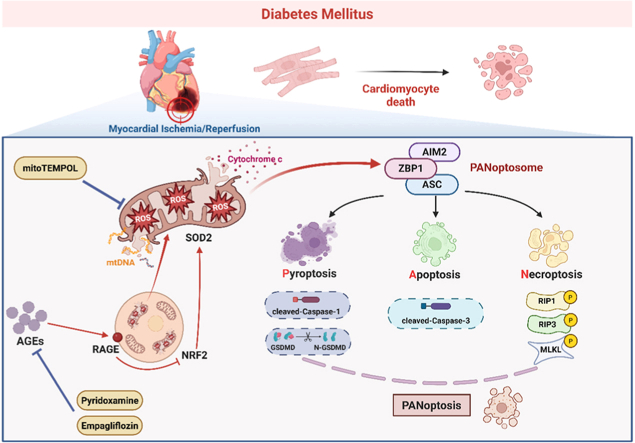

## Abbreviations

AAR: area at risk
AGEs: advanced glycation end-products
AIM2: absent in melanoma 2
AMI: acute myocardial infarction
ANOVA: analysis of variance
ASC: apoptosis-associated speck-like protein containing a CARD
ATP: adenosine triphosphate
BCA: bicinchoninic acid
BSA: bovine serum albumin
cTnT: cardiac troponin T
DM: diabetes mellitus
DNA: deoxyribonucleic acid
DMEM: dulbecco's modified eagle medium
ECL: enhanced chemiluminescence
FBS: fetal bovine serum
FS: fractional shortening
GSDMD: gasdermin D
H/R: hypoxia/reoxygenation
HRP: horseradish peroxidase
IA: infarct area
IHC: immunohistochemistry
LDH: lactate dehydrogenase
LVEF: left ventricular ejection fraction
MI/RI: myocardial ischemia/reperfusion injury
MI/R: myocardial ischemia/reperfusion
mtDNA: mitochondrial deoxyribonucleic acid
mtROS: mitochondrial reactive oxygen species
NLRP3: NOD-like receptor thermal protein domain associated protein 3
Nrf2: nuclear factor erythroid 2-related factor 2
OGD: oxygen–glucose deprivation
PBS: phosphate-buffered saline
PI: propidium iodide
*p*-MLKL: phosphorylated mixed lineage kinase like domain
p-RIP1: phosphorylated receptor-interacting protein 1
p-RIP3: phosphorylated receptor-interacting protein 3
PVDF: polyvinylidene difluoride
PyriEMPA: pyridoxamine and empagliflozin
RAGE: receptor for AGEs
SD: standard deviation
SGLT2: sodium-glucose cotransporter 2
SOD2: superoxide dismutase 2
SPF: specific pathogen-free
TBST: tris-buffered saline containing 0.1% tween-20
TEM: transmission electron microscopy
TSA: tyramide signal amplification
TTC: 2,3,5-triphenyltetrazolium chloride
WB: western blot
ZBP1: Z-DNA binding protein 1
ΔΨm: mitochondrial membrane potential

## 1 Introduction

Acute myocardial infarction (AMI) remains the leading cause of cardiovascular mortality worldwide, despite substantial advances in pharmacological and interventional therapies [1]. Myocardial ischemia/reperfusion injury (MI/RI) occurs following revascularization of the occluded vessel, contributing to expanded infarct size and compromised cardiac function after AMI, thereby increasing the risk of in-hospital adverse cardiovascular events [2,3]. Diabetes mellitus (DM) is the most prevalent comorbidities in AMI patients, currently accounting for over one-third of all AMI cases [4]. It is well established that DM elevates the risk of AMI due to its pro-atherosclerotic and pro-thrombotic effects [[5], [6], [7]]. Numerous clinical studies have shown that DM is strongly associated with higher rates of cardiovascular death, heart failure, and arrhythmias in patients hospitalized for AMI [[8], [9], [10], [11]]. Nevertheless, the precise influence of DM on MI/RI and its underlying pathological mechanisms remain incompletely elucidated.

Advanced glycation end-products (AGEs) are a heterogeneous family of compounds formed via non-enzymatic glycation of proteins, lipids, and nucleic acids by reducing sugars, primarily glucose [12]. Endogenous AGEs accumulate under conditions of hyperglycemia and are closely linked to diabetes and its complications [[13], [14], [15]]. however, their effect on MI/RI and the associated molecular mechanisms have not been thoroughly investigated. PANoptosis is a pro-inflammatory programmed cell death that integrates components and key features of apoptosis, pyroptosis, and necroptosis. It is initiated by the assembly of a multifaceted protein complex called the PANoptosome [[16], [17], [18]]. Emerging evidence implicates PANoptosis in the pathogenesis of various inflammatory and infectious diseases [[19], [20], [21]]. In the cardiovascular realm, its involvement in septic cardiomyopathy, atherosclerosis, and AMI has been reported [22]. However, the upstream triggers and regulatory mechanisms of PANoptosis in the diabetic heart, and specifically its potential link to metabolic insults like AGEs, remain entirely unexplored.

Current standard-of-care for patients with diabetes and AMI prioritizes timely revascularization, coupled with guideline-directed medical therapy including antiplatelet agents, statins, beta-blockers, and renin-angiotensin-aldosterone system inhibitors [1]. While these strategies have improved outcomes, a significant residual risk of heart failure and mortality persists in the diabetic population [23]. This underscores the existence of distinct, incompletely targeted pathological pathways that are particularly active in the diabetic heart. One such pathway is the formation and accumulation of AGEs. Although the detrimental role of AGEs in microvascular complications is recognized, there are currently no clinically approved therapeutic strategies specifically designed to mitigate AGEs-mediated injury in the setting of AMI. Given this unmet need, the present study investigated the combination therapy of pyridoxamine, a well-characterized AGEs-formation inhibitor that has reached clinical trials for diabetic nephropathy [24,25], and empagliflozin, an SGLT2 inhibitor with proven cardioprotective benefits in large outcome trials [26]. This strategy is distinct from current clinical care and directly targets a pathway implicated in diabetic vulnerability to MI/RI.

In this study, we aimed to validate the impact of DM on MI/RI, examine the contribution of AGEs to this process, and elucidate the underlying mechanisms using both in vivo and in vitro models. Additionally, we proposed and evaluated a novel therapeutic strategy to attenuate diabetes-aggravated MI/RI in animal experiments.

## 2 Methods

### 2.1 Animals

BKS-db/db mice, a well-established spontaneous diabetic model, were used in this study, with BKS-db/m mice serving as non-diabetic controls. All mice were purchased from SPF (Beijing) Biotechnology Co., Ltd. and housed under specific pathogen-free (SPF) conditions at Zhongshan Hospital. Animal care and experimental procedures were conducted in compliance with the guidelines from Directive 2010/63/EU of the European Parliament on the protection of animals used for scientific purpose and approved by the Animal Care and Use Committee of Zhongshan Hospital, Fudan University (Approved No.: 2025-103). The ethical approval was obtained prior to the initiation of any animal work. The duration of the animal experiments was 4-8 weeks, which falls well within the validity period of the ethical approval (15/4/2025-30/5/2026). The experimental timeline was designed to ensure sufficient time for diabetic phenotype development, drug intervention, surgery, and post-operative observation. At the end of each experiment, mice were deeply anesthetized via inhalation of 2% isoflurane and then euthanized by cervical dislocation. Cardiac tissues were harvested immediately for subsequent experiments.

### 2.2 Myocardial ischemia/reperfusion (MI/R) surgery

Prior to surgery, baseline parameters including body weight and blood glucose levels were recorded. Diabetic and non-diabetic mice were randomly assigned to either MI/R surgery or sham surgery groups. Under anesthesia induced by inhalation of 2% isoflurane, a left thoracic incision was performed in mice to expose the heart. Myocardial ischemia was induced by ligating the left anterior descending coronary artery with a 6-0 silk suture (Surgical Specialties Corporation, UK) for 40 min, followed by reperfusion via release of the suture. Sham-operated mice underwent identical procedures without coronary artery ligation.

### 2.3 Drug intervention

Pyridoxamine dihydrochloride (#545068, Sigma-Aldrich, USA) and empagliflozin (#HY-15409, MedChemExpress, China) were administered prior to MI/R surgery. Mice were randomly assigned to receive either a combination of pyridoxamine and empagliflozin (PyriEMPA group), pyridoxamine alone (Pyri group), empagliflozin alone (EMPA group), or saline as a control (Saline group). Pyridoxamine was administered via drinking water at a dosage of 100 mg/kg/day. Empagliflozin (10 mg/kg/day) was delivered by intraperitoneal injection. Control mice received equivalent volumes of saline intraperitoneally without pyridoxamine supplementation. Drug treatment commenced one month before surgery and continued throughout the experimental period.

### 2.4 Echocardiography

Following anesthesia induction with 2% isoflurane (inhaled), cardiac structure and function were assessed 24 h after MI/R surgery using transthoracic echocardiography (VisualSonics VeVo 2100 Imaging System, Canada). Body temperature was maintained between 36.9 and 37.3 °C, and heart rates were stabilized at 400–500 beats per minute. Left ventricular ejection fraction (LVEF) and fractional shortening (FS) were calculated from M-mode echocardiographic recordings.

### 2.5 TTC and Evans Blue staining

At 24 h post-MI/R, mice were anesthetized via inhalation of 2% isoflurane, and the left anterior descending coronary artery was re-occluded. Evans blue dye (1%; #E2129, Sigma-Aldrich, USA) was injected retrogradely via the aorta to demarcate the non-ischemic area (blue). Following Evans Blue perfusion, hearts were excised, rinsed in ice-cold PBS, and briefly blotted dry. The ventricles were then cut perpendicular to the long axis into 4-5 slices of approximately 1.5-2.0 mm in thickness. The slices were immediately incubated in a pre-warmed 1% solution of 2,3,5-triphenyltetrazolium chloride (TTC; #T8877, Sigma-Aldrich, USA) in PBS (pH 7.4) for 30 min at 37 °C in the dark. After incubation, the slices were gently rinsed with PBS and fixed by immersion in 10% neutral buffered formalin for a minimum of 4 h at 4 °C to enhance the contrast between stained (viable, red) and unstained (necrotic, pale) tissue. The fixed slices were then arranged and imaged. The area at risk (AAR) was defined as tissue unstained by Evans blue, and the infarct area (IA) as tissue unstained by TTC. The images were analyzed using Fiji (ImageJ) software.

### 2.6 Masson staining

Heart tissues harvested 24 h after MI/R surgery were fixed in 10% neutral buffered formalin, embedded in paraffin, and sectioned at 4–5 μm thickness. After deparaffinization and hydration, sections were stained with Weigert's iron hematoxylin for nuclei and picrosirius red for collagen, followed by dehydration and mounting. Collagen fibers appeared blue, muscle fibers red, and nuclei dark blue/black under high-resolution microscopy.

### 2.7 TSA-based multiplex immunofluorescent staining

Deparaffinized heart sections underwent antigen retrieval and endogenous blocking before incubation with primary antibodies (4 °C overnight or 37 °C for 1–2 h). HRP-conjugated secondary antibodies were applied, followed by tyramide signal amplification (TSA) with fluorophores. Iterative rounds of staining were performed for multiple targets. Slides were mounted and imaged using spectral confocal microscopy. Antibody and fluorophore details are listed in Supplementary Table 1.

### 2.8 Cell culture

H9C2 cardiomyocytes (#ZQ0102, Shanghai Zhong Qiao Xin Zhou Biotechnology Co., Ltd.) were maintained in high-glucose Dulbecco's Modified Eagle Medium (DMEM) supplemented with 10% fetal bovine serum (FBS), 100 U/mL penicillin, and 100 μg/mL streptomycin at 37 °C in a humidified atmosphere of 5% CO_2_. Cells were routinely passaged at 80-90% confluence, typically every 2 days. Briefly, the culture medium was removed, and cells were washed once with 1X phosphate-buffered saline (PBS). Subsequently, cells were detached using 0.25% trypsin-EDTA solution (incubated at 37 °C for 2-3 min), followed by neutralization with complete growth medium. The cell suspension was then centrifuged at 200 × g for 5 min. The pellet was resuspended in fresh complete medium, and cells were seeded into new culture vessels at a split ratio of 1:2.

### 2.9 Hypoxia/reoxygenation (H/R)

At 80–90% confluence, H9C2 cells were subjected to oxygen–glucose deprivation (OGD) by replacing medium with deoxygenated glucose-free DMEM (#11966-025, Thermo Fisher Scientific, USA) and incubating in a hypoxic chamber (94% N_2_, 1% O_2_, 5% CO_2_) for 12 h. Reoxygenation was initiated by returning cells to complete DMEM under normoxic conditions for 4 h. Based on preliminary experiments, 12h-hypoxia induced substantial metabolic stress and sensitized cells to reoxygenation injury, while keeping baseline necrosis during hypoxia itself at a moderate level (<20%). This created a “primed” state analogous to the vulnerable myocardium in ischemia. 4h-reoxygenation captured the critical early reperfusion injury phase, providing a suitable window to study the mechanistic cascade from mitochondrial oxidative damage to PANoptosis.

### 2.10 Chemical treatments

To establish a chronic model of AGEs exposure mimicking the diabetic milieu, cells were cultured in medium supplemented with 100 μM AGEs (#22968, Cayman Chemical) for five consecutive passages prior to H/R experiments. This extended priming period was designed to allow for the intracellular accumulation of AGEs and the potential induction of adaptive or maladaptive cellular responses. The AGEs-containing medium was maintained throughout the entire priming period and was also present during the subsequent H/R stimulation, ensuring continuous exposure. Control cells were cultured in parallel in standard medium without AGEs supplementation for the same number of passages. To induce mitochondrial ROS (mtROS) burst and mitochondrial oxidative damage, naive H9C2 cardiomyocytes (without AGEs priming and H/R stimulation) were treated with 7.5 μM antimycin A (#A8647, Sigma-Aldrich, Germany) for 1 h. To blocked mitochondrial oxidative damage, MitoTEMPOL (#HY-115386, MedChemExpress, China) was added at 10 μM upon H/R initiation and maintained thereafter in the complete medium.

### 2.11 siRNA transfection

H9C2 cardiomyocytes were seeded in antibiotic-free medium to reach 60-70% confluence. For each well of a 6-well plate, 5 μL of 20 μM siRNA targeting receptor for AGEs (*RAGE*), absent in melanoma 2 (*Aim2)*, Z-DNA binding protein 1 (*Zbp1)*, nuclear factor erythroid 2-releated factor 2 (*Nrf2)*, or a non-targeting control (Supplementary Table 2) was mixed with 250 μL Opti-MEM (#31985-062, Thermo Fisher Scientific, USA). Separately, 2.5 μL Hieff Trans® liposomal 2000 transfection reagent (#40802ES03, Yeasen Biotech, China) was diluted in 250 μL Opti-MEM. After 5 min, the two solutions were combined, incubated for 20 min at room temperature, and added dropwise to the cells. The transfection mixture was replaced with complete medium after 6 h. Knockdown efficiency was validated 48-72 h post-transfection by Western blotting.

### 2.12 Real-time cell imaging

Cell death was monitored using propidium iodide (PI; #P3566, Thermo Fisher Scientific, USA) at 0.5 mg/mL and Hoechst 33342 (#C1025, Beyotime Biotech, China) at 10 μg/mL. Images were acquired every 30 min using an Agilent BioTek Gen5 system, and the ratio of PI-positive to Hoechst-positive cells was quantified.

### 2.13 Detection of mtROS

MtROS levels were quantified using the mitochondrial-targeted, superoxide-sensitive fluorescent probe MitoSOX Red (#36008, Thermo Fisher Scientific, USA). Following the designated treatments, H9C2 cardiomyocytes were washed with warm PBS and incubated with 5 μM MitoSOX Red, 100 nM MitoTracker Green (#1996 M, Beyotime Biotech, China), and 10 μg/mL Hoechst 33342 in serum-free medium for 15 min at 37 °C in the dark. MitoTracker Green and Hoechst 33342 were co-stained for mitochondrial and nuclear localization, respectively. After incubation, cells were washed three times with warm PBS. For confocal microscopy, cells were immediately imaged using a standard FITC/Texas Red filter set. For flow cytometry, cells were trypsinized, resuspended in PBS, and analyzed on a flow cytometer. MitoSOX Red fluorescence (excitation/emission: 510/580 nm) was used to quantify mtROS levels.

### 2.14 Mitochondrial membrane potential assay

Mitochondrial membrane potential (ΔΨm) was assessed using the JC-1 assay kit (#C2003S, Beyotime Biotech, China) following the manufacturer's protocol. Briefly, H9C2 cardiomyocytes, seeded in either black-walled 96-well plates (for quantification) or confocal dishes (for imaging), were treated as indicated. Following experimental treatments, cells were washed once with pre-warmed PBS and then incubated with JC-1 working solution (prepared by diluting the stock solution 1:100 in serum-free, dye-free medium) for 20 min at 37 °C in a 5% CO_2_ incubator, protected from light. After incubation, cells were washed twice with pre-warmed JC-1 staining buffer. For quantitative analysis, fluorescence was immediately measured using a microplate reader (Varioskan LUX, Thermo Fisher Scientific, USA). JC-1 aggregates, indicative of high ΔΨm, were detected at excitation/emission wavelengths of 525/590 nm, while JC-1 monomers, indicative of low ΔΨm, were detected at 490/530 nm. The ΔΨm was expressed as the ratio of aggregate (red) to monomer (green) fluorescence intensity. For imaging, cells were visualized immediately under a confocal microscope using identical laser settings across all samples. Representative images from at least three independent experiments are shown.

### 2.15 ATP assay

Intracellular ATP content was quantified using an Enhanced ATP Assay Kit (#C0027, Beyotime Biotech, China) according to the manufacturer's protocol. Briefly, after experimental treatments, H9C2 cardiomyocytes were lysed using the provided lysis buffer. The lysate was centrifuged at 12,000 × g for 5 min at 4 °C. Equal volumes of supernatant and ATP detection working solution were combined in an opaque 96-well plate. Luminescence was immediately measured using a microplate reader. ATP concentrations were determined by comparison to a concurrently run standard curve of known ATP concentrations and normalized to the total protein concentration of each sample.

### 2.16 Cytosolic mitochondrial DNA (mtDNA) extraction

The cytosolic fraction used for mtDNA analysis was obtained using a well-established digitonin-based subcellular fractionation kit (#C360, Beyotime Biotech, China). The absence of significant mitochondrial contamination in the cytosolic fraction was confirmed by Western blotting for cytochrome *c*. Following fractionation, DNA from the cytosolic fraction was isolated using a column-based kit (#18700 ES, Yeasen Biotech, China). To control for potential nuclear DNA contamination, qPCR primers that specifically amplify a region of the mitochondrial genome (*mtCOI*) and a region of the nuclear genome (*18S rRNA*) were designed (Supplementary Table 2). The amount of cytosolic mtDNA was calculated using the standard curve method and is expressed relative to the nuclear DNA content in the same sample. Only samples with negligible amplification of the nuclear gene in the cytosolic fraction (Ct value > 30 or undetectable) were included in the final analysis for mtDNA release.

### 2.17 Transmission electron microscopy (TEM)

Heart tissues and cardiomyocytes were fixed in 2.5% glutaraldehyde, post-fixed in 1% osmium tetroxide, dehydrated in ethanol, and embedded in epoxy resin. Ultrathin sections (70–90 nm) were stained with 2% phosphotungstic acid and imaged using a Hitachi HT7800 microscope at 80 kV.

### 2.18 Western blotting (WB)

Total protein was extracted from H9C2 cardiomyocytes or frozen left ventricular tissues. The protein concentration was determined using a bicinchoninic acid (BCA) protein assay kit (#P0012, Beyotime Biotech, China), with bovine serum albumin (BSA) as the standard. Equal amounts of protein (20 μg) were loaded onto SDS-polyacrylamide gels for electrophoretic separation. Following electrophoresis, proteins were transferred from the gel onto polyvinylidene difluoride (PVDF) membranes (#88518, Millipore, USA) using a wet transfer system. After transfer, the membranes were blocked for 1 h at room temperature with 5% BSA and then incubated overnight at 4 °C with specific primary antibodies. Then the membranes were incubated with horseradish peroxidase (HRP)-conjugated secondary antibodies for 1 h at room temperature. Protein bands were visualized using enhanced chemiluminescence (ECL) substrate and imaged with a chemiluminescence imaging system. Band intensities were quantified using the gel analysis function in Fiji (ImageJ) software. Information of antibodies is provided in Supplementary Table 1.

### 2.19 Statistical analysis

The sample size (n) for each experiment represents the number of biologically independent animals or cell culture replicates. For animal experiments, the group size for surgical procedures (MI/R or Sham) was determined based on a priori power analysis and our laboratory's historical data, which indicated that n = 5-6 per group is typically sufficient to detect significant differences in primary endpoints in this model. Differences in sample size between assays for the same animal cohort occur because some assays require the entire tissue sample, precluding its use for other measurements. while other non-destructive assays can be performed on all animals, potentially resulting in a larger ‘n'. In vitro experiments involving H9C2 cardiomyocytes were performed with biological replicates on at least three independent occasions. Each independent experiment began with the thawing of a new vial of cells from cell bank, followed by cell culture, treatment, and endpoint analysis. The data points presented in graphs are pooled from multiple, temporally separated experimental runs, not from multiple wells within a single plate on a single day. The biological replicate for cell-based assays was an independent culture well/dish. Technical replicates were averaged to yield a single data point per independent experiment. The exact “n” number, representing the count of biological replicates. Data are expressed as mean ± SD for normally distributed continuous variables. Unpaired *t*-test was used to compare between two independent groups. Multiple groups were compared using one-way or two-way ANOVA with post hoc tests (Tukey's or Sidak's). A p-value <0.05 was considered statistically significant. All analyses were performed using GraphPad Prism 8.0.

## 3 Results

### 3.1 Diabetes mellitus exacerbates myocardial ischemia/reperfusion injury

**Fig. 1:**
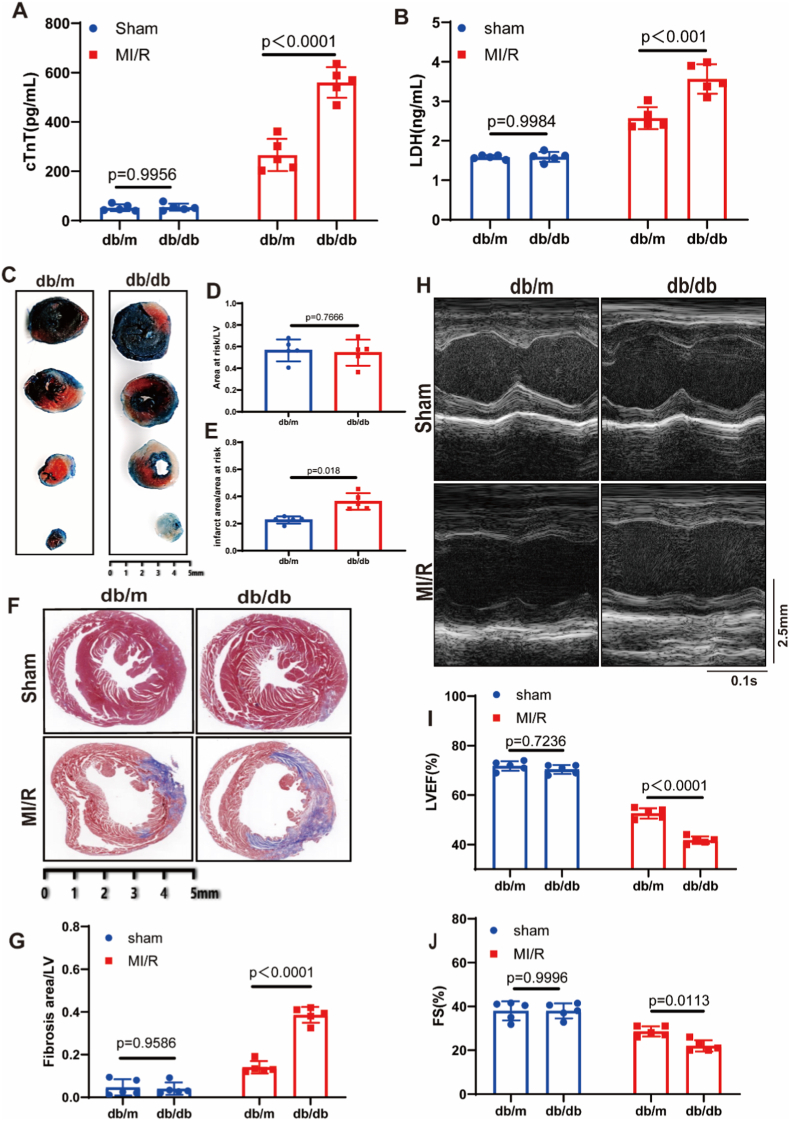
**Diabetes mellitus exacerbates myocardial ischemia/reperfusion injury.** **(A)** Serum levels of cardiac troponin T (cTnT). **(B)** Serum levels of lactate dehydrogenase (LDH). **(C)** Representative images of TTC/Evans Blue staining in diabetic (db/db) and non-diabetic (db/m) mice. Scale bar: 5 mm. **(D)** Quantitative analysis of the area at risk (AAR) normalized to left ventricular (LV) area. **(E)** Quantitative analysis of infarct area (IA) normalized to AAR. **(F)** Representative images of Masson staining. Scale bar: 5 mm. **(G)** Quantitative analysis of fibrotic area normalized to LV area. **(H)** Representative M-mode echocardiographic images. Time stamp: 0.1s; Scale bar: 2.5 mm. **(I)** Left ventricular ejection fraction (LVEF) measured by echocardiography. **(J)** Fractional shortening (FS) measured by echocardiography. n = 5 in each group. Data were presented as mean ± SD. (A, B, I, G, J) *P*-value was produced using two-way ANOVA followed by Sidak's multiple comparisons test. (D, E) *P*-value was calculated by unpaired Student's *t*-test. A two-tailed *P* < 0.05 was considered statistically significant.

To investigate the impact of DM on MI/RI, diabetic db/db mice and non-diabetic db/m mice (8–10 weeks old) were randomly subjected to MI/R or sham surgery. Baseline body weight and blood glucose levels were recorded prior to surgery (Supplementary Fig. 1A and B). Serum levels of myocardial injury markers, including cardiac troponin T (cTnT) and lactate dehydrogenase (LDH), were significantly elevated in diabetic mice (Fig. 1A and B). TTC/Evans blue staining revealed a markedly larger myocardial infarct size in diabetic mice despite a similar area at risk (AAR) between diabetic and non-diabetic groups (Fig. 1C–E). Masson staining indicated more severe myocardial fibrosis in diabetic mice (Fig. 1F and G). Echocardiography demonstrated impaired left ventricular function in diabetic mice, reflected by reduced left ventricular ejection fraction (LVEF) and fractional shortening (FS) compared with non-diabetic controls (Fig. 1H–J). Together, these results confirm that DM aggravates MI/RI.

### 3.2 Advanced glycation end-products promote PANoptosis in cardiomyocytes after MI/R under diabetic conditions

**Fig. 2:**
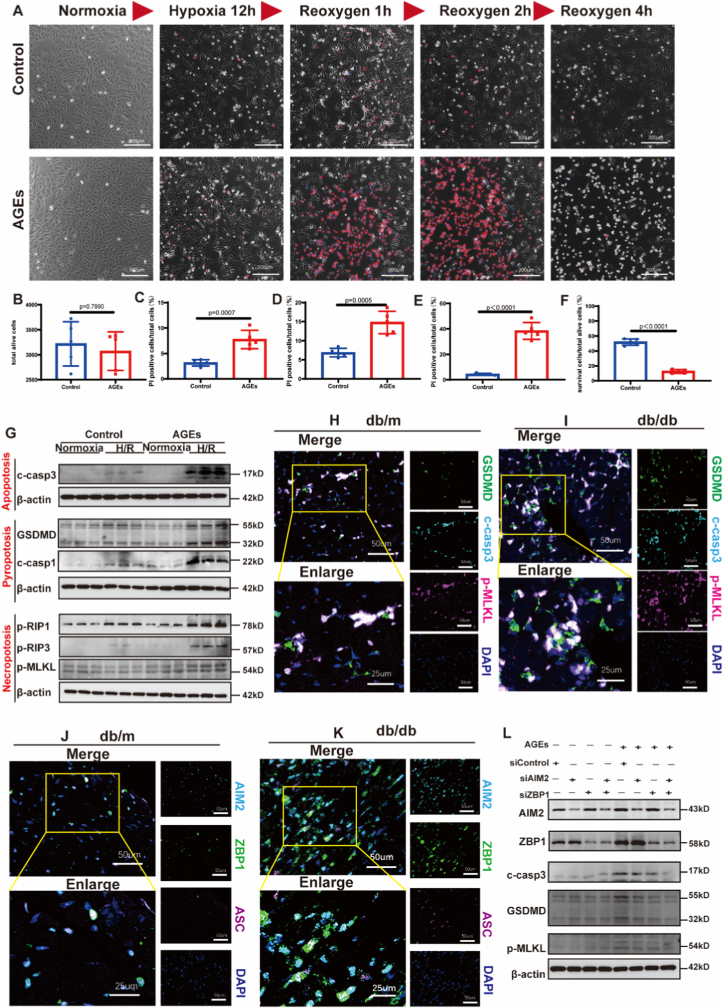
**Advanced glycation end-products promote PANoptosis in cardiomyocytes after myocardial ischemia/reperfusion**. **(A)** Real-time imaging of cardiomyocyte death during hypoxia/reoxygenation (H/R). scale bar: 300 μm. **(B)** Total number of viable cardiomyocytes under normoxic conditions. **(C)** Proportion of PI-positive cells after 12 h of hypoxia. **(D, E)** Proportion of PI-positive cells at 1 and 2 h after reoxygenation, respectively. **(F)** Cell survival rate expressed as the percentage of viable cells relative to baseline after 4 h of reoxygenation. **(G)** Western blot analysis of key proteins associated with apoptosis (cleaved-caspase3), pyroptosis (cleaved-caspase1, GSDMD), and necroptosis (p-RIP1, p-RIP3, *p*-MLKL). **(H, I)** Immunofluorescence staining of GSDMD, cleaved-caspase3, and *p*-MLKL in heart tissues from diabetic (db/db) and non-diabetic (db/m) mice 24 h after MI/R. **(J, K)** Immunofluorescence colocalization of AIM2, ZBP1, and ASC, demonstrating assembly of the AIM2-ZBP1-PANoptosome in heart tissues from diabetic (db/db) and non-diabetic (db/m) mice 24 h after MI/R. **(L)** Western blot analysis of PANoptosis executors in cardiomyocytes following genetic ablation of AIM2, ZBP1, or both. c-casp3: cleaved caspase-3. c-casp1: cleaved caspase-1. Scale bars: 50 μm (H–K). n (biological replicates) = 5 in each group. Data were presented as mean ± SD. (B–F) Statistical analyses were conducted by unpaired Student's *t*-test. A two-tailed *P* < 0.05 was considered statistically significant.

AGEs, accumulating during prolonged hyperglycemia, were significantly elevated in the serum and heart tissue of diabetic mice (Supplementary Fig. 1C–G). To evaluate the role of AGEs in MI/RI, cardiomyocytes were cultured in AGEs-supplemented medium for five passages; control cells were maintained in standard medium. No significant differences in cellular morphology or differentiation rate were observed between AGEs-treated and control groups under normoxia (Fig. 2A and B). However, after 12 h of hypoxia, AGEs-primed cardiomyocytes showed heightened susceptibility to cell death, as indicated by increased PI staining. Real-time cell imaging further revealed sustained cell death following reoxygenation in the AGEs-treated group (Fig. 2A–C–E). Nearly 90% of AGEs-treated cells underwent cell death within 4 h after reoxygenation, whereas control cells exhibited only mild cell death and began recovering by 2 h post-reoxygenation (Fig. 2A, B, F).

**Fig. 3:**
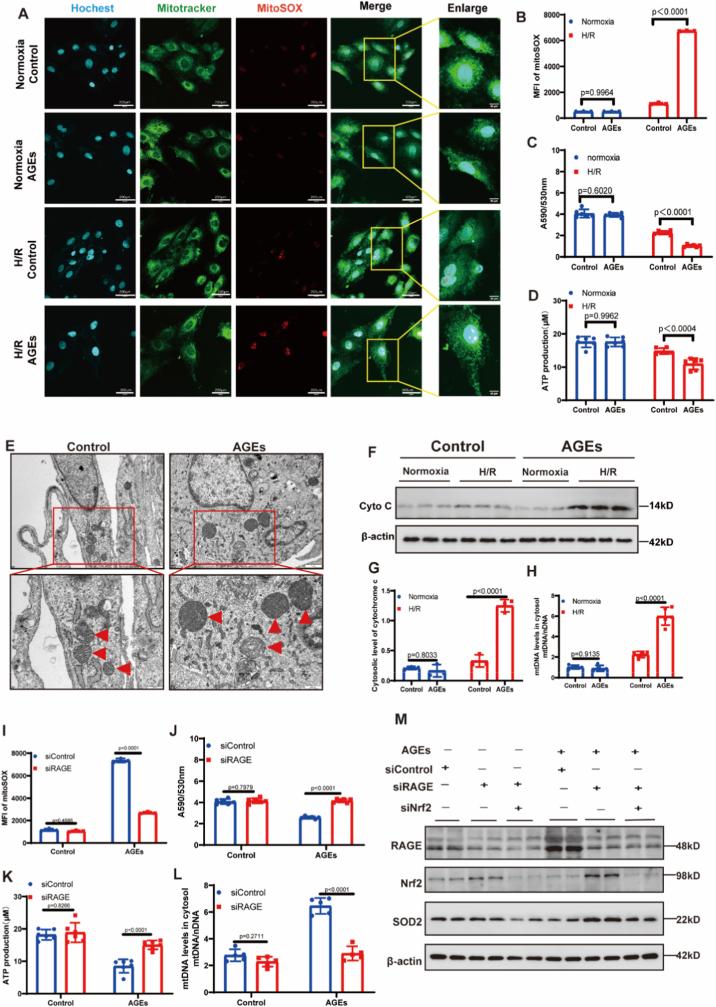
**Advanced glycation end-products exacerbate mitochondrial oxidative damage upon hypoxia/reoxygenation stimulation**. **(A)** Representative images of MitoSOX staining showing mitochondrial ROS levels in cardiomyocytes after H/R stimulation. Scale bar: 200 μm. **(B)** Quantitative analysis of MitoSOX mean fluorescence intensity (MFI) by flow cytometry, n (biological replicates) = 3. **(C)** Mitochondrial membrane potential assessed by the JC-1 fluorescence ratio (590/530 nm), n (biological replicates) = 5. **(D)** Cellular ATP production after H/R stimulation, n (biological replicates) = 5. **(E)** Transmission electron microscopy images of cardiomyocytes cultured with AGEs-containing or control medium; red arrows indicate mitochondria. Scale bar: 1μm/500 nm(enlarged). **(F)** Representative western blots of cytochrome *c* in cytosol. **(G)** Quantitative analysis of cytosolic cytochrome *c* levels normalized to β-actin, n (biological replicates) = 3. **(H)** Quantitative analysis of cytosolic mtDNA, n (biological replicates) = 5. **(I)** Quantitative analysis (mean fluorescence intensity, MFI) of mtROS in H9C2 cardiomyocytes by flow cytometry using MitoSOX staining, following RAGE knockdown, n (biological replicates) = 3. **(J)** Mitochondrial membrane potential assessed by the JC-1 fluorescence ratio (590/530 nm), following RAGE knockdown, n (biological replicates) = 5. **(K)** Cellular ATP production after H/R stimulation, following RAGE knockdown, n (biological replicates) = 5. **(L)** Quantitative analysis of cytosolic mtDNA, following RAGE knockdown, n (biological replicates) = 5. **(M)** Western blot analysis of mitochondrial superoxide dismutase (SOD2) in cardiomyocytes following genetic ablation of *RAGE* alone, or dual ablation of *RAGE* and *Nrf2*. Data were presented as mean ± SD. (B, C, D, G, H, I, J, K, L) *P*-value was produced using two-way ANOVA followed by Sidak's multiple comparisons test. A two-tailed *P* < 0.05 was considered statistically significant.

At the molecular level, proteins associated with multiple cell death pathways were upregulated in AGEs-treated cardiomyocytes after 2 h of reoxygenation. Specifically, apoptosis-related cleaved caspase-3, pyroptosis-related cleaved caspase-1 and GSDMD, and necroptosis-associated p-RIP1, p-RIP3, and *p*-MLKL were all elevated (Fig. 3G; Supplementary Fig. 2A–F), indicating activation of PANoptosis. Consistent with these in vitro findings, cleaved caspase-3, GSDMD, and *p*-MLKL were also significantly increased in heart tissues from diabetic mice 24 h after MI/R compared with non-diabetic controls (Fig. 2H and I). The assembly of PANoptosome is initial step for PANoptosis. Several proteins, including AIM2, ZBP1, and NLRP3, have been reported to assemble into PANoptosomes to initiate PANoptosis. In the present study, AIM2 and ZBP1 were significantly upregulated (Supplementary Fig. 2G–I) and exhibited enhanced colocalization with ASC in the infarcted myocardium of diabetic mice (Fig. 2J and K). In contrast, NLRP3 was barely detectable in cardiomyocytes (Supplementary Fig. 2G).

To further validate the involvement of the AIM2-ZBP1 PANoptosome in AGEs-induced PANoptosis, loss-of-function experiments were performed using specific siRNAs targeting *Aim2* and *Zbp1*. Efficient knockdown of AIM2 or ZBP1was confirmed (Supplementary Fig. 2J–M). Notably, silencing either AIM2 or ZBP1 alone significantly attenuated PANoptosis in AGEs-primed cardiomyocytes, as evidenced by reduced expression of key PANoptosis executers; Dual knockdown of AIM2 and ZBP1 completely abolished PANoptosis, demonstrating a synergistic requirement for both sensors in this integrated cell death pathway (Fig. 2L; Supplementary Fig. 2N–P). These results establish AIM2 and ZBP1 as essential and non-redundant components of the PANoptosome that mediates AGEs-induced PANoptosis.

### 3.3 Advanced glycation end-products exacerbate mitochondrial oxidative damage upon H/R stimulation

Given the established role of mitochondrial damage in triggering PANoptosis under oxidative stress, we first assessed mtROS levels in AGEs-treated cardiomyocytes following H/R. Under normoxic conditions, MitoSOX staining revealed comparably low mtROS levels in both AGEs-treated and control cells. However, upon H/R stimulation, AGEs-treated cells exhibited significantly elevated mtROS (Fig. 3A and B; Supplementary Fig. 3A). Consistent with oxidative stress, JC-1 staining demonstrated a pronounced decline in ΔΨm in AGEs-primed cardiomyocytes after H/R, indicating severe mitochondrial dysfunction (Fig. 3C; Supplementary Fig. 3B). This mitochondrial impairment was further reflected by a marked reduction in ATP production (Fig. 3D). Notably, TEM revealed no obvious morphological differences in mitochondria between AGEs-treated and control cells under normoxia (Supplementary Fig. 3C). In contrast, following H/R, AGEs-treated cardiomyocytes showed distinct mitochondrial swelling and cristae disruption (Fig. 3E). Moreover, the cytosolic release of mitochondrial components, including cytochrome *c* and mtDNA, was significantly increased in AGEs-treated cells after H/R (Fig. 3F–H), indicating substantial mitochondrial oxidative damage in AGEs-primed cardiomyocytes.

RAGE, the primary receptor for AGEs, was significantly upregulated in AGEs-primed cardiomyocytes (Supplementary Fig. 3D). To investigate the functional contribution of RAGE signaling to cardiomyocyte vulnerability, we performed loss-of-function studies by transfecting cells with RAGE-specific siRNA prior to AGEs priming. Efficient knockdown of RAGE was confirmed (Supplementary Fig. 3F and G). Importantly, RAGE silencing markedly attenuated the mitochondrial oxidative damage triggered by subsequent H/R in AGEs-primed cells, as evidenced by reduced mtROS, preserved ΔΨm, and restored ATP levels (Fig. 3I–L). We next examined the potential mechanism linking RAGE to mitochondrial redox imbalance. Both Nrf2, the central transcriptional regulator of the antioxidant response, and its key mitochondrial target-superoxide dismutase 2 (SOD2)-were significantly downregulated in AGEs-treated cardiomyocytes (Supplementary Fig. 3H–J). To determine whether AGEs impair mitochondrial antioxidant defense via a RAGE–Nrf2–SOD2 axis, we conducted combinatorial genetic interventions. Silencing RAGE effectively restored the expression of Nrf2 and SOD2. Conversely, co-silencing of Nrf2 abolished the beneficial effect of RAGE knockdown on SOD2 expression (Fig. 3M, Supplementary Fig. 3K–M). These data establish a sequential signaling cascade in which AGEs, acting through RAGE, suppress the Nrf2–SOD2 pathway, thereby compromising mitochondrial antioxidant capacity and sensitizing cardiomyocytes to oxidative injury.

### 3.4 Mitochondrial oxidative damage mediates AGEs-Induced PANoptosis

**Fig. 4:**
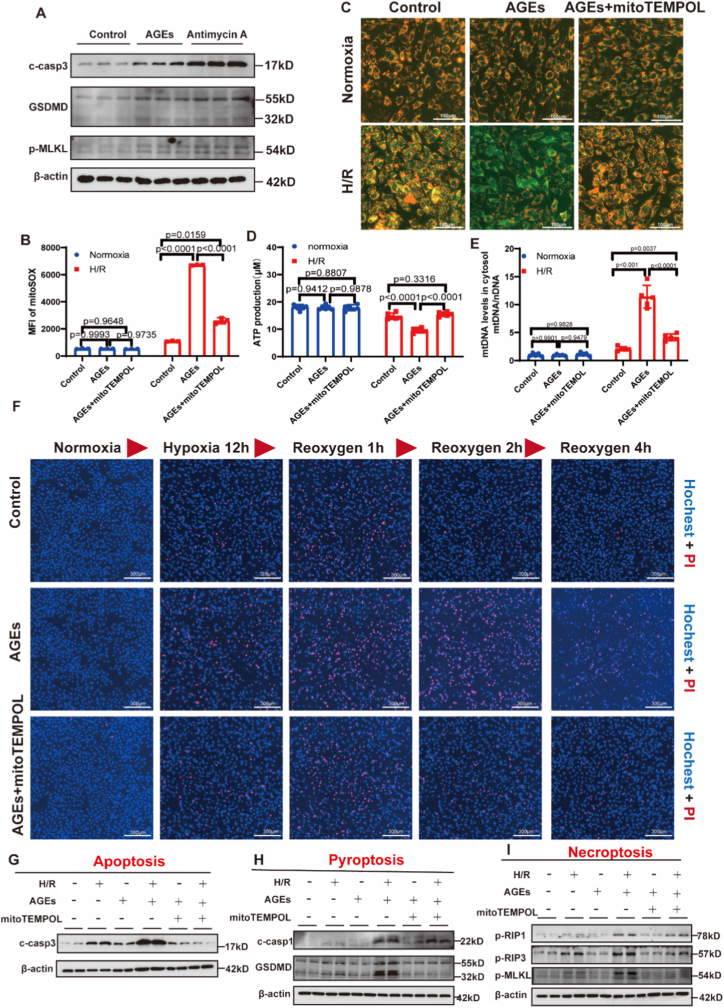
Mitochondrial Oxidative Damage mediates AGEs-induced PANoptosis. **(A)** Western blot analysis of PANoptosis executors (cleaved caspase-3, GSDMD, and *p*-MLKL) in H9C2 cardiomyocytes treated with the mitochondrial oxidative stress inducer antimycin A or AGEs. **(B)** Quantitative analysis of MitoSOX mean fluorescence intensity (MFI), n (biological replicates) = 3. **(C)** Representative confocal microscopy images of JC-1 staining (scale bar: 100 μm). **(D)** ATP production in cardiomyocytes after H/R stimulation, n (biological replicates) = 5. **(E)** mtDNA levels in cytosol of cardiomyocytes after H/R stimulation, n (biological replicates) = 5. **(F)** Real-time monitoring of cardiomyocyte death during H/R (scale bar: 300 μm). **(G)** Western blot analysis of apoptosis marker cleaved caspase-3. **(H)** Western blot analysis of pyroptosis markers cleaved caspase-1 and GSDMD. **(I)** Western blot analysis of necroptosis markers p-RIP1, p-RIP3, and *p*-MLKL. c-casp3: cleaved caspase-3. c-casp1: cleaved caspase-1. Data were presented as mean ± SD. (B, D, E) *P*-value was produced using one-way ANOVA followed by Tukey's multiple comparisons test. A two-tailed *P* < 0.05 was considered statistically significant.

To directly assess whether mitochondrial oxidative damage is sufficient to trigger PANoptosis, we treated cardiomyocytes with antimycin A, a specific inhibitor of mitochondrial complex III, to induce a rapid mtROS burst and mitochondrial oxidative damage. Compared to vehicle-treated controls, antimycin A robustly activated PANoptosis, as evidenced by a marked upregulation of key PANoptosis executers (Fig. 4A; Supplementary Fig. 4A–C). Notably, the extent of PANoptosis activation induced by antimycin A was comparable to that elicited by AGEs priming upon H/R stimulation, indicating that direct mitochondrial oxidative insult is sufficient to drive PANoptosis.

To further determine whether mitochondrial oxidative damage specifically mediates AGEs-induced PANoptosis, we pre-treated AGEs-primed cardiomyocytes with the mitochondria-targeted antioxidant MitoTEMPOL prior to H/R. MitoTEMPOL significantly attenuated mitochondrial injury, as shown by reduced mtROS levels (Fig. 4B; Supplementary Fig. 4D), preserved ΔΨm (Fig. 4C; Supplementary Fig. 4E), and restored ATP production (Fig. 4D). Moreover, MitoTEMPOL suppressed the cytosolic release of cytochrome *c* and mtDNA (Fig. 4E; Supplementary Fig. 4F and G). Consistent with mitochondrial protection, MitoTEMPOL treatment markedly reduced H/R-induced cell death (Fig. 4F; Supplementary Fig. 4H–L) and downregulated the expression of PANoptosis executers (Fig. 4G–I; Supplementary Fig. 4M–R).

To exclude potential off-target effects of the pharmacological antioxidant, we employed a genetic approach to endogenously enhance mitochondrial antioxidant capacity. Silencing of RAGE in AGEs-primed cardiomyocytes restored SOD2 expression (Supplementary Fig. 4S and T), thereby reducing mtROS via an endogenous mechanism. Importantly, similar to MitoTEMPOL treatment, RAGE silencing significantly attenuated the activation of PANoptosis (Supplementary Fig. 4U–W), confirming that the reduction of mtROS—whether by pharmacological scavenging or by boosting endogenous antioxidant defense—is sufficient to suppress PANoptosis.

Taken together, these complementary pharmacological and genetic interventions demonstrate that mitochondrial oxidative damage acts as an essential upstream trigger and a critical mediator of AGEs-induced PANoptosis in cardiomyocytes.

### 3.5 Pyridoxamine and empagliflozin alleviate AGEs-induced mitochondrial damage and PANoptosis

**Fig. 5:**
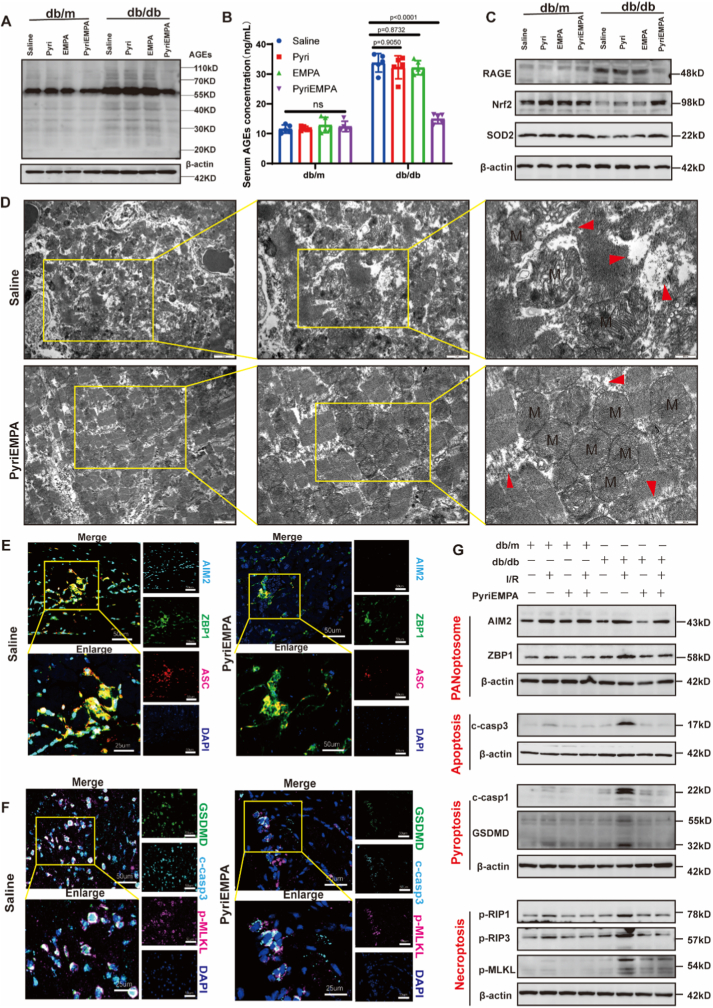
**Treatment with pyridoxamine and empagliflozin attenuates AGE-induced mitochondrial damage and PANoptosis**. **(A)** Representative Western blot analysis of AGE levels in heart tissues from non-diabetic (db/m), diabetic (db/db) mice receiving different treatments. **(B)** Quantitative analysis of AGE expression normalized to β-actin, n (biological replicates) = 3. **(C)** Western blot analysis of RAGE, Nrf2, SOD2 in heart tissues from non-diabetic (db/m), diabetic (db/db) mice receiving different treatments. **(D)** Transmission electron microscopy images of myocardial ultrastructure in diabetic mice treated with saline or PyriEMPA. M: mitochondria; red arrows indicate sarcomeric damage. Scale bar: 2μm/1μm/500 nm. **(E)** Immunofluorescence colocalization of AIM2, ZBP1, and ASC, indicating AIM2-ZBP1-PANoptosome assembly. Scale bar: 50 μm. **(F)** Immunofluorescence staining of GSDMD, cleaved caspase-3, and *p*-MLKL in heart sections from saline- or PyriEMPA-treated diabetic mice. Scale bar: 50 μm. **(G)** Western blot analysis of AIM2-ZBP1 PANoptome and PANoptosis executors. c-casp3: cleaved caspase-3. c-casp1: cleaved caspase-1. Data were presented as mean ± SD. (B) *P*-value was generated using one-way ANOVA followed by Tukey's multiple comparisons test. A two-tailed *P* < 0.05 was considered statistically significant.

Given the detrimental role of AGEs in diabetic myocardial injury, we next evaluated whether pharmacological inhibition of AGEs formation could attenuate MI/RI. Although pyridoxamine has been reported to inhibit AGEs formation, its efficacy is often limited under persistent hyperglycemic conditions. We therefore hypothesized that combining pyridoxamine with the glucose-lowering agent empagliflozin would more effectively suppress AGE accumulation and its downstream effects. To verify this hypothesis, diabetic mice were treated with a combination of pyridoxamine and empagliflozin (PyriEMPA) for one month prior to MI/R surgery. Body weight and blood glucose levels were recorded before surgery (Supplementary Fig. 5A and B). PyriEMPA pretreatment significantly reduced serum AGE levels and cardiac AGE accumulation compared with either monotherapy (Fig. 5A and B; Supplementary Fig. 5C). At the molecular level, PyriEMPA pretreatment suppressed the expression of RAGE and restored mitochondrial antioxidant defense axis, as evidenced by upregulated levels of Nrf2 and its downstream effector SOD2 in diabetic hearts (Fig. 5A; Supplementary Fig. 5D–F).

Consistent with these findings, TEM analysis revealed that diabetic mice exhibited severe mitochondrial and sarcomeric damage, including mitochondrial swelling, cristae fragmentation, and myofibrillar disarray, 24 h post-MI/R, whereas PyriEMPA-pretreated mice maintained near-normal ultrastructure (Fig. 5D; Supplementary Fig. 5G). Moreover, PyriEMPA treatment suppressed AIM2-ZBP1 PANoptosome activation and downregulated PANoptosis executors (Fig. 5E–G; Supplementary Fig. 5H–M).

Together, these results demonstrate that combined inhibition of AGEs formation and glycemic control by PyriEMPA effectively attenuates mitochondrial oxidative damage and PANoptosis activation in diabetic hearts.

### 3.6 Combination therapy with pyridoxamine and empagliflozin improves MI/RI outcomes in diabetic mice

**Fig. 6:**
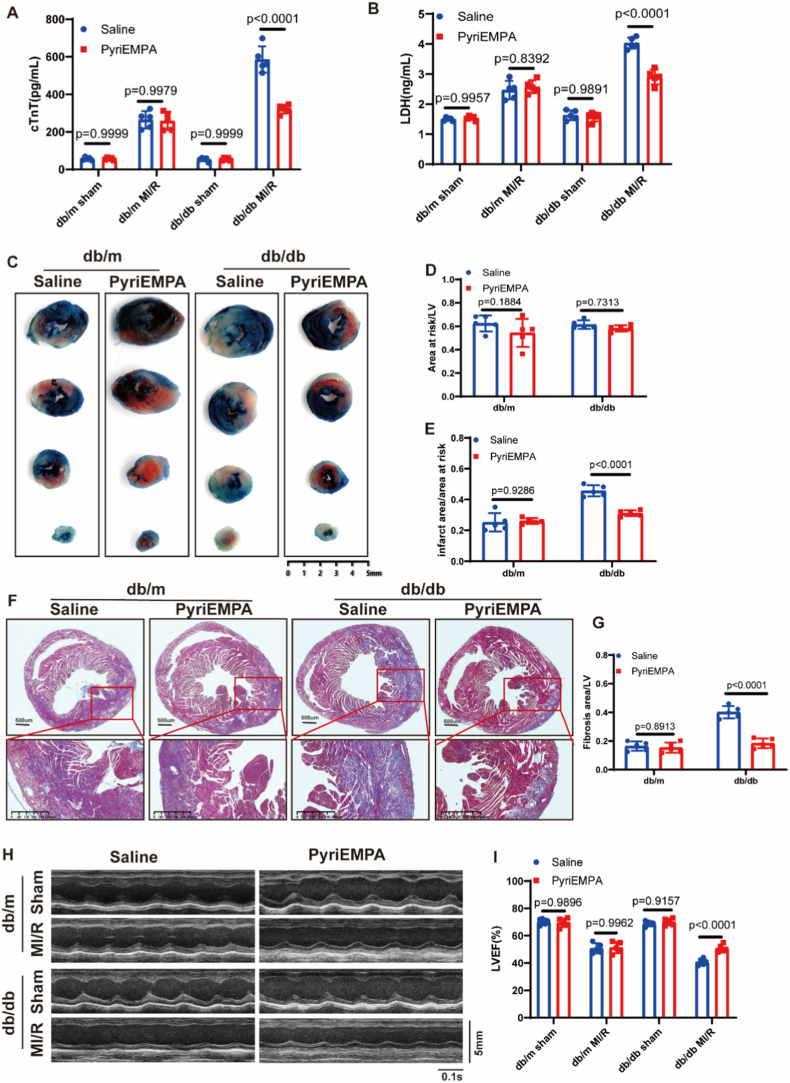
Combination therapy with pyridoxamine and empagliflozin improve MI/RI outcomes in diabetic mice. **(A)** Serum levels of cardiac troponin T (cTnT). **(B)** Serum levels of lactate dehydrogenase (LDH). **(C)** Representative images of TTC/Evans Blue staining in diabetic (db/db) and non-diabetic (db/m) mice. Scale bar: 5 mm. **(D)** Quantitative analysis of the area at risk (AAR) normalized to left ventricular (LV) area. **(E)** Quantitative analysis of infarct area (IA) normalized to AAR. **(F)** Representative images of Masson staining. Scale bar: 500μm/300 μm. **(G)** Quantitative analysis of fibrotic area normalized to LV area. **(H)** Representative M-mode echocardiographic images. Time stamp: 0.1s; Scale bar: 5 mm. **(I)** Left ventricular ejection fraction (LVEF) measured by echocardiography. n (the number of animals used) = 5 in each group. Data were presented as mean ± SD. (A, B, I) *P*-value was calculated using one-way ANOVA followed by Tukey's multiple comparisons test. (D, E, G) *P*-value was calculated using two-way ANOVA followed by Sidak's multiple comparisons test. A two-tailed *P* < 0.05 was considered statistically significant.

Finally, we evaluated the therapeutic efficacy of PyriEMPA in vivo. Compared with saline-treated diabetic controls, PyriEMPA-pretreated mice exhibited significantly lower serum levels of cTnT and LDH (Fig. 6A and B), reduced infarct size (Fig. 6C–E), attenuated myocardial fibrosis (Fig. 6F and G), and improved cardiac function, as reflected by higher LVEF (Fig. 6H and I). These results indicate that PyriEMPA pretreatment alleviates MI/RI, mitigates adverse cardiac remodeling, and promotes functional recovery in diabetic mice post-MI/R.

Although empagliflozin monotherapy has previously been reported to exert cardioprotective effects, neither pyridoxamine nor empagliflozin alone attenuated mitochondrial oxidative damage or PANoptosis in the present study (Supplementary Fig. A–F). However, empagliflozin monotherapy did improve myocardial function compared with saline or pyridoxamine treatment, suggesting that its protective mechanisms operate independently of the *AGEs–mitochondrial damage–PANoptosis* axis. Notably, the combination of pyridoxamine and empagliflozin resulted in more pronounced functional improvement than empagliflozin alone (Supplementary Fig. 6G).

Taken together, these findings demonstrate that combination therapy with pyridoxamine and empagliflozin synergistically improves MI/RI outcomes in diabetic mice.

## 4 Discussion

The global prevalence of DM has increased markedly in recent decades, and projections indicate that combined pre-diabetes and DM will affect half of the global population by 2050, posing a substantial public health challenge [27]. DM is a well-established risk factor for AMI, primarily by accelerating atherosclerosis and promoting thrombosis [7,10]. Consequently, the rising incidence of DM directly contributes to an elevated AMI burden. More critically, once AMI occurs, DM is strongly associated with worse clinical outcomes, including higher in-hospital mortality, reinfarction rates, heart failure, cardiogenic shock, and arrhythmias [[28], [29], [30], [31]]. This distinct, aggravated clinical trajectory strongly suggests that DM fundamentally alters the pathophysiology of post-ischemic myocardial injury. While the epidemiological association is clear, the precise molecular mechanisms by which DM exacerbates MI/RI remained incompletely elucidated. Our study provides direct in vivo evidence that diabetic mice subjected to MI/R develop more severe injury, characterized by elevated serum biomarkers, larger infarct size, enhanced fibrosis, and worse cardiac function compared to non-diabetic controls. These data confirm the detrimental impact of the diabetic milieu on MI/RI.

Among the myriad metabolic disturbances in DM, the accumulation of AGEs represents a hallmark of chronic hyperglycemia [12,13,15]. The deleterious effects of AGEs are multifaceted, including the cross-linking of structural proteins and, critically, the activation of cellular signaling via RAGE, driving oxidative stress and chronic inflammation [[32], [33], [34], [35]]. While the role of AGEs in microvascular complications and atherosclerosis is recognized, their specific contribution to MI/RI in diabetes had not been established. In the present study, we demonstrate that AGEs, by engaging RAGE, initiate a signaling cascade that suppresses the nuclear activity of the master antioxidant transcription factor Nrf2 [[36], [37], [38]]. This suppression leads to the downregulation of SOD2, the primary enzyme responsible for detoxifying superoxide anions within the mitochondrial matrix, serving as the first line of defense against mtROS [[38], [39], [40], [41]]. The functional consequence of this “RAGE-Nrf2-SOD2″ axis disruption is a profound weakening of the intrinsic mitochondrial antioxidant capacity. Consequently, upon H/R stimulation, AGEs-primed cardiomyocytes are incapable of handling the sudden mtROS burst, leading to an exacerbated and uncontrolled accumulation of mtROS. This oxidative surge directly damages mitochondrial components, manifesting as loss of membrane potential, impaired ATP synthesis, and the release of mtDNA and cytochrome *c*. The pathological significance of the mtROS burst extends beyond mitochondrial damage. Upon H/R stimulation, we observed the specific upregulation and co-localization of AIM2 and ZBP1 with the adaptor protein ASC in AGEs-primed cardiomyocytes, indicating the assembly of an AIM2-ZBP1 PANoptosome. Pharmacological scavenging of mtROS or genetic enhancement of mitochondrial antioxidant defense suppressed AIM2-ZBP1 PANoptosome assembly and the subsequent execution of PANoptosis. Conversely, directly inducing mitochondrial oxidative stress with antimycin A was sufficient to trigger PANoptosis, independent of AGEs. This bidirectional evidence provides robust proof that mitochondrial oxidative damage acts as a necessary and sufficient upstream trigger for PANoptosome activation and PANoptosis, which is in align with previous literatures [[42], [43], [44]].

Given the established detrimental role of AGEs in exacerbating MI/RI, they represent a critical and promising therapeutic target for intervention. Previous studies indicate that pyridoxamine can inhibit AGE formation in diabetes, however, its clinical efficacy was constrained under persistent hyperglycemia [24,25]. In the present study, we evaluated the co-administration of pyridoxamine and empagliflozin, an SGLT2 inhibitor with proven cardiometabolic benefits beyond glucose lowering [26,45,46]. This combination, termed PyriEMPA, was designed to simultaneously target the causative factor (hyperglycemia/metabolic dysfunction via empagliflozin) and a key downstream effector (AGEs via pyridoxamine). Our results demonstrate that PyriEMPA pretreatment in diabetic mice significantly reduced cardiac AGEs accumulation, restored the RAGE-Nrf2-SOD2 axis, preserved mitochondrial function, suppressed the AIM2-ZBP1 PANoptosome, and ultimately attenuated infarct size and improved cardiac function after MI/R. The efficacy of PyriEMPA surpassed that of either agent alone, supporting a synergistic mechanism wherein empagliflozin improves the metabolic milieu, thereby enhancing and sustaining the therapeutic effect of pyridoxamine. This highlights the critical importance of multi-targeted strategies in diabetic complications and presents PyriEMPA as a promising, readily translatable therapeutic regimen for diabetic patients at high risk of AMI.

Despite the comprehensive nature of our investigation, certain limitations warrant acknowledgment. First, while our in vitro model effectively isolated the direct effects of pre-formed AGEs, future studies employing a combined chronic high-glucose and AGE exposure system would more fully recapitulate the ongoing diabetic metabolic insult and its potential synergy with AGEs. Second, our in vitro data demonstrate that exogenous AGEs alone are sufficient to sensitize cardiomyocytes to H/R injury. This raises the intriguing possibility, testable in future studies, that exogenous AGE administration could exacerbate MI/RI even in a non-diabetic model, which would further solidify AGEs as a standalone therapeutic target for MI/RI. Finally, while the preclinical efficacy of PyriEMPA is compelling, its therapeutic potential in diabetic patients with MI/RI ultimately requires validation in randomized controlled clinical trials.

## Funding

The work was supported by Fund from 10.13039/501100009076University of Science and Technology of China (KY9100000023 to junbo Ge) and the 10.13039/501100001809National Natural Science Foundation of China (82470334 to Feng Zhang, 82000333 to Ji'e Yang).

## CRediT authorship contribution statement

**Ji'e Yang:** Conceptualization, Formal analysis, Funding acquisition, Investigation, Methodology, Project administration, Writing – original draft. **Jian Zhang:** Conceptualization, Data curation, Investigation, Methodology, Resources, Software. **Rong Huang:** Conceptualization, Data curation, Investigation, Methodology, Resources, Software. **Ke Meng:** Investigation, Methodology, Resources. **Jiayu Liang:** Investigation, Methodology, Methodology, Investigation. **Jingyi Lin:** Investigation, Methodology. **Jingpu Wang:** Investigation, Methodology. **Yiweng Wang:** Investigation, Methodology. **Yang Gao:** Methodology. **Yanan Qu:** Methodology. **Shiyu Hu:** Methodology. **Jiatian Cao:** Methodology. **Hao Su:** Conceptualization, Resources, Supervision, Writing – review & editing. **Feng Zhang:** Conceptualization, Funding acquisition, Resources, Supervision, Writing – review & editing. **Junbo Ge:** Conceptualization, Funding acquisition, Supervision, Writing – review & editing.

## Declaration of competing interest

The authors declare that they have no known competing financial interests or personal relationships that could have appeared to influence the work reported in this paper.

## Data Availability

The datasets used and/or analyzed in the current study are available from the corresponding author on reasonable request.
