# Supplementary material for: Advanced glycation end-products exacerbate myocardial ischemia/reperfusion injury by promoting mitochondrial oxidative damage and PANoptosis in diabetes mellitus
Source: Redox Biol. 2026 May 20;94:104228. doi: 10.1016/j.redox.2026.104228 (PMC13224011; doi:10.1016/j.redox.2026.104228)
Supplement: Multimedia component 1 [file mmc1.doc]

**Supplementary Fig.1**


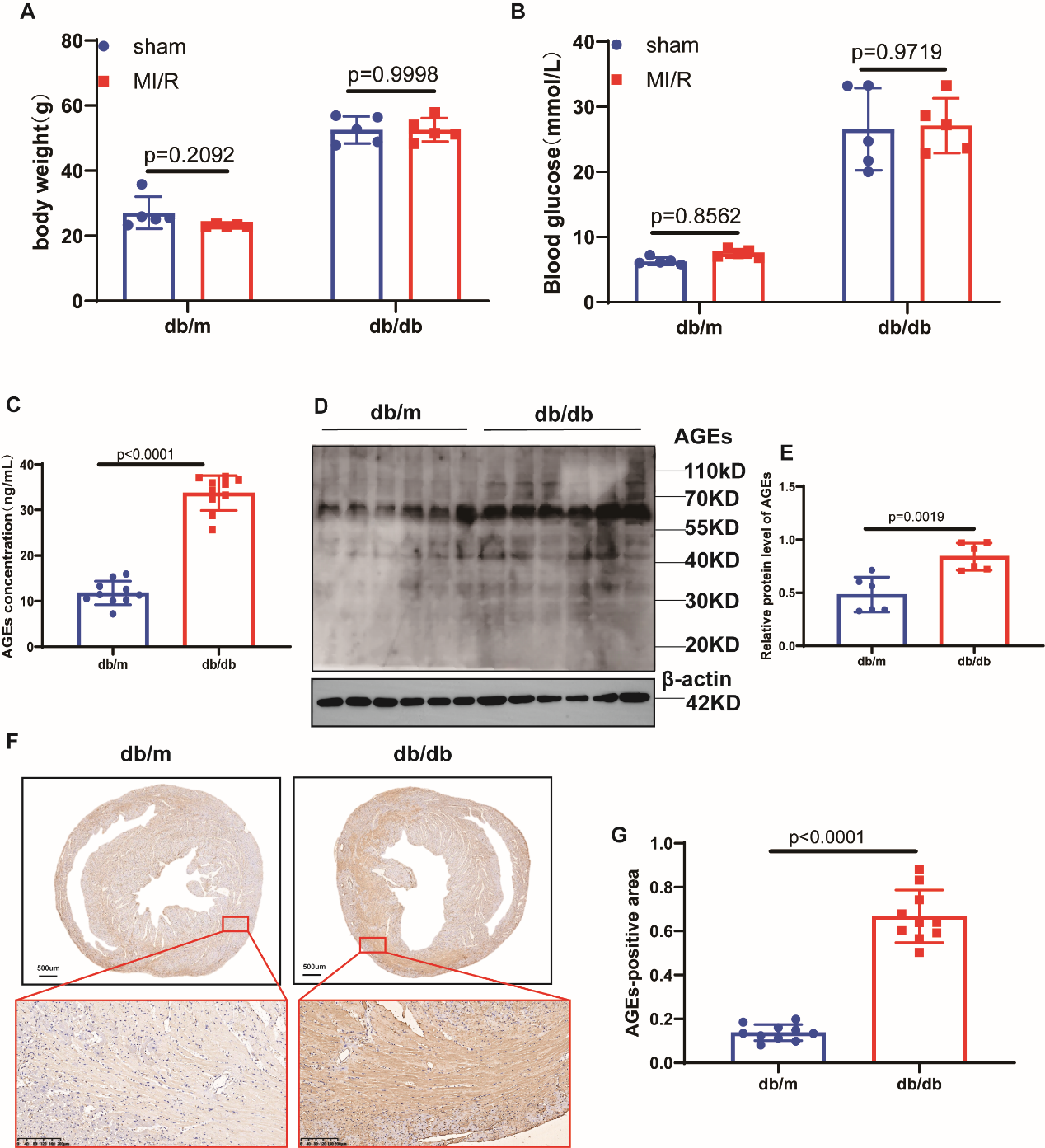


**(A)** Baseline body weight of db/db (diabetic) and db/m (non-diabetic) mice (n=5). **(B)** Baseline blood glucose levels (n=5). **(C)** Serum levels of advanced glycation end-products (AGEs)(n=10). **(D)** Representative Western blot analysis of AGEs expression in heart tissue. **(E)** Quantitative analysis of AGEs protein levels normalized to β-actin (n=6). **(F)** Representative immunohistochemical staining of AGEs in myocardial sections. Scale bar: 500μm/200μm. **(G)** Quantitative analysis of AGEs-positive area (n=10). Data were presented as mean ± SD. (A, B) *P*-value was calculated using two-way ANOVA followed by Sidak’s multiple comparisons test. (C, E, G) *P*-value was calculated by unpaired Student’s *t*-test. A two-tailed *P* < 0.05 was considered statistically significant.

**Supplementary Fig.2**


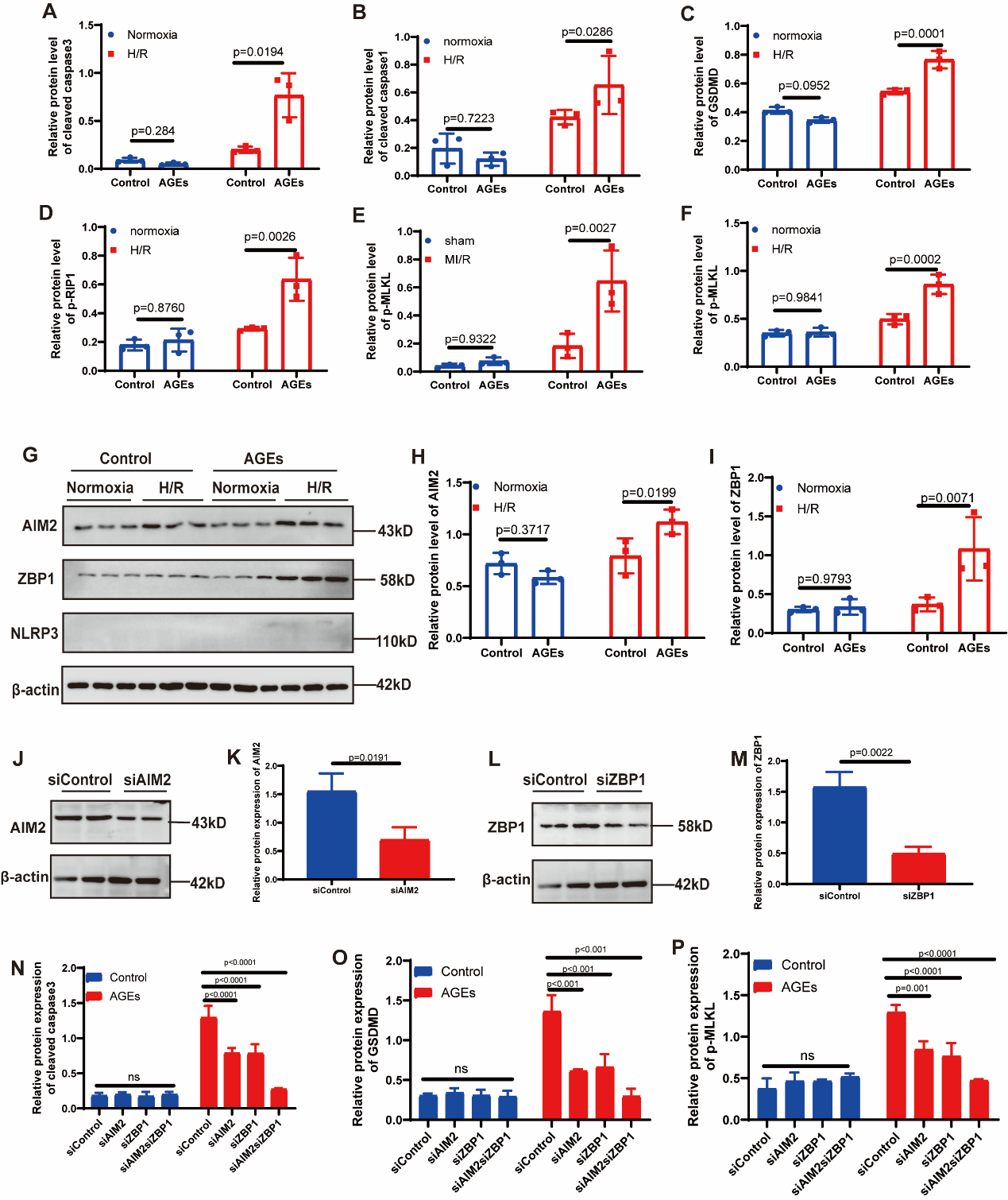


Quantitative analysis of relative protein expression of **(A)** cleaved caspase-3, **(B)** cleaved caspase-1, **(C)** GSDMD, **(D)** p-RIP1, **(E)** p-RIP3, and **(F)** p-MLKL. **(G)** Representative western blots of AIM2, ZBP1, NLRP3. Quantitative analysis of relative protein expression of **(H)** AIM2, **(I)** ZBP1 in the indicated groups. Western blot verification of **(J)** AIM2 and **(L)** ZBP1 silencing in cardiomyocytes. Quantitative analysis of relative protein expression of **(K)** AIM2 and **(M)** ZBP1 in cardiomyocytes transfected with indicated siRNA. Quantitative analysis of relative protein expression of **(N)** cleaved caspase3, **(O)** GSDMD**, (P)** p-MLKL in the indicated groups. n (biological replicates) =3 in each group. Data were presented as mean ± SD. (A-F, H, I) *P*-value was calculated using two-way ANOVA followed by Sidak’s multiple comparisons test. (K, M) *P*-value was calculated using unpaired Student’s t test. (N-P) *P*-value was calculated using one-way ANOVA followed by Tukey’s multiple comparisons test. A two-tailed *P* < 0.05 was considered statistically significant.

**Supplementary Fig.3**


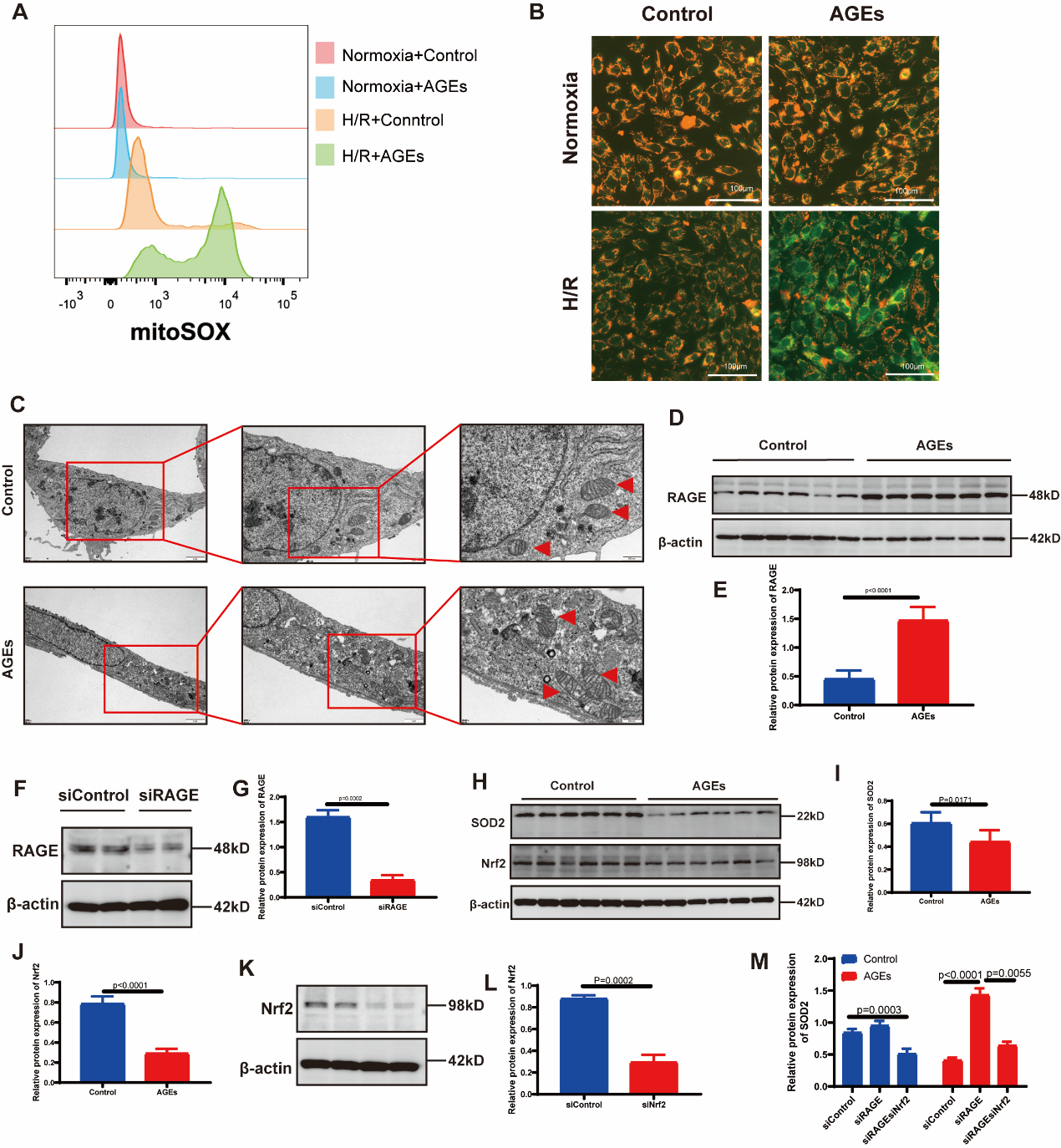


**(A)** Representative flow cytometry histograms showing MitoSOX Red fluorescence intensity. **(B)** Representative confocal microscopy images of JC-1 staining in cardiomyocytes (scale bar: 200μm). **(C)** Transmission electron microscopy images of cardiomyocytes cultured in AGE-containing or control medium under normoxia (scale bar: 2μm/1μm/500nm). Representative Western blot analysis of **(D)** RAGE, **(H)**SOD2 and Nrf2 expression in cardiomyocytes cultured in normal or AGEs-contained medium. Quantitative analysis of relative protein expression of **(E)** RAGE, **(I)** SOD2, and **(J)** Nrf2 in the indicated groups. Western blot verification of **(F)** RAGE and **(K)** Nrf2 silencing in cardiomyocytes. Quantitative analysis of relative protein expression of **(G)** RAGE and **(L)** Nrf2 in cardiomyocytes transfected with indicated siRNA. Quantitative analysis of relative protein expression of **(M)** SOD2 in the indicated groups. (E, G, I, J, L) *P*-value was calculated using unpaired Student’s t test. (M) *P*-value was calculated using two-way ANOVA followed by Turkey’s multiple comparisons test.

**Supplementary Fig. 4**


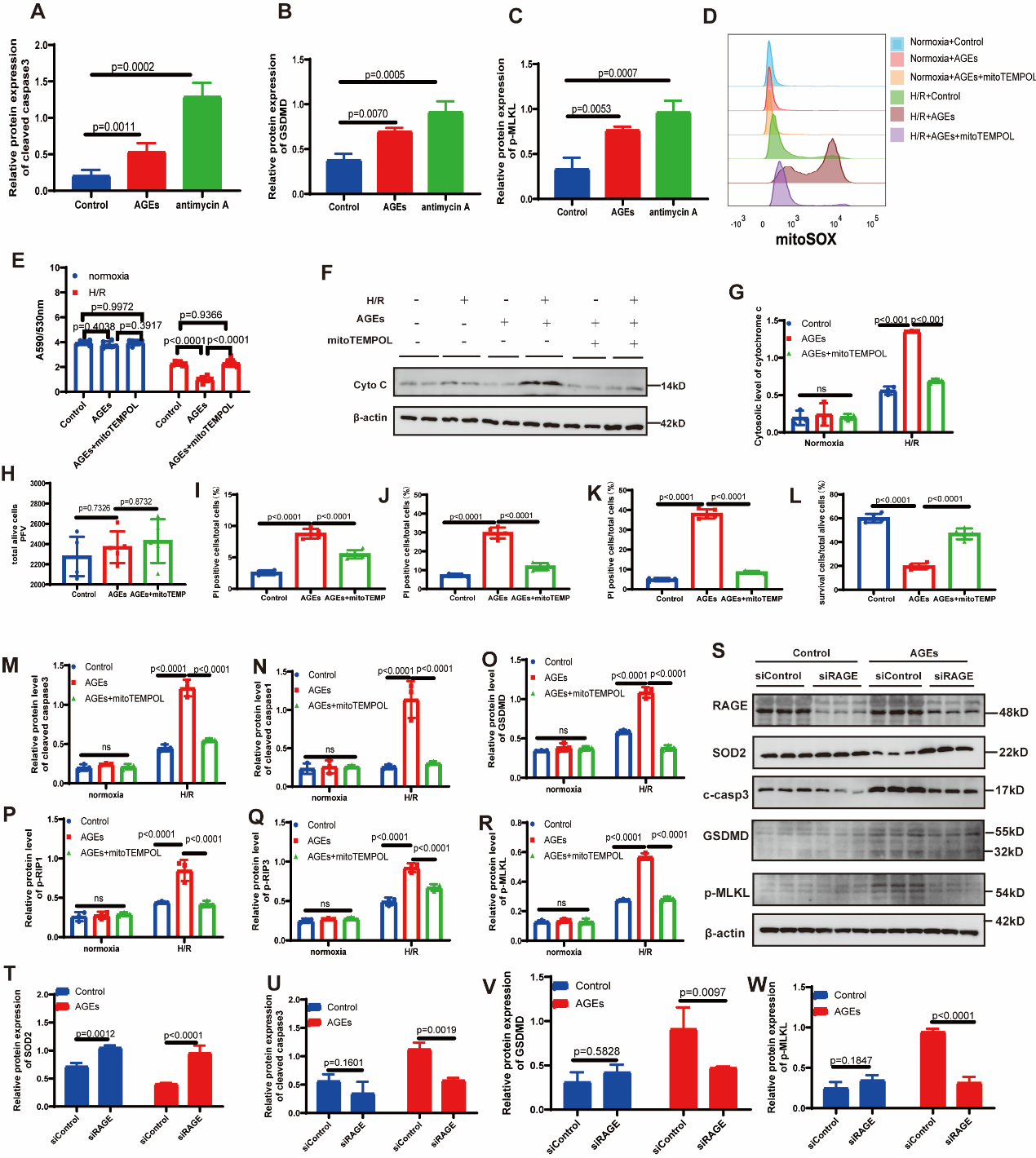


​​Quantitative analysis of relative protein expression levels of **​​**​​**(A)**​​ cleaved caspase-3, **​​(B)**​​ GSDMD, and ​​**(C)​​** p-MLKL. (D) Representative histograms of MitoSOX fluorescence intensity measured by flow cytometry. **(E)** Mitochondrial membrane potential assessed by the JC-1 fluorescence ratio (590/530 nm), n (biological replicates) =5**. (F)** Representative western blots of mitochondrial cytosolic c released in cytosol. **(G)** Quantitative analysis of relative cytochrome c in cytosol normalized to β-actin. **(H)​​** Total number of viable cardiomyocytes under normoxic conditions. **​​(I)**​​ Proportion of PI-positive cells after 12 hours of hypoxia. **​​(J, K)**​​ Proportion of PI-positive cells at 1 and 2 hours after reoxygenation, respectively. **​​(L)**​​ Cell survival rate expressed as the percentage of viable cells relative to baseline after 4 hours of reoxygenation. Quantitative analysis of relative protein expression levels of **​​(M)​​** cleaved caspase-3, ​​**(N)**​​ cleaved caspase-1, **​​(O)**​​ GSDMD, **​​(P)​​** p-RIP1, ​​**(Q)​​** p-RIP3, and ​​**(R)​​** p-MLKL. **(S)** Representative western blots of SOD2 and PANoptosis executors in RAGE silencing or control cardiomyocytes. Quantitative analysis of relative protein expression levels of **​​(T)​​** SOD2, ​​**(U)**​​ cleaved caspase-3, **​​(V)**​​ GSDMD, and ​​**(W)​​** p-MLKL. (A-C, E, G, H-R) *P*-value was produced using one-way ANOVA followed by Tukey’s multiple comparisons test. (T-W) *P*-value was calculated using two-way ANOVA followed by Sidak’s multiple comparisons test. Data were presented as mean ± SD. A two-tailed *P* < 0.05 was considered statistically significant.

**Supplementary Fig.5**


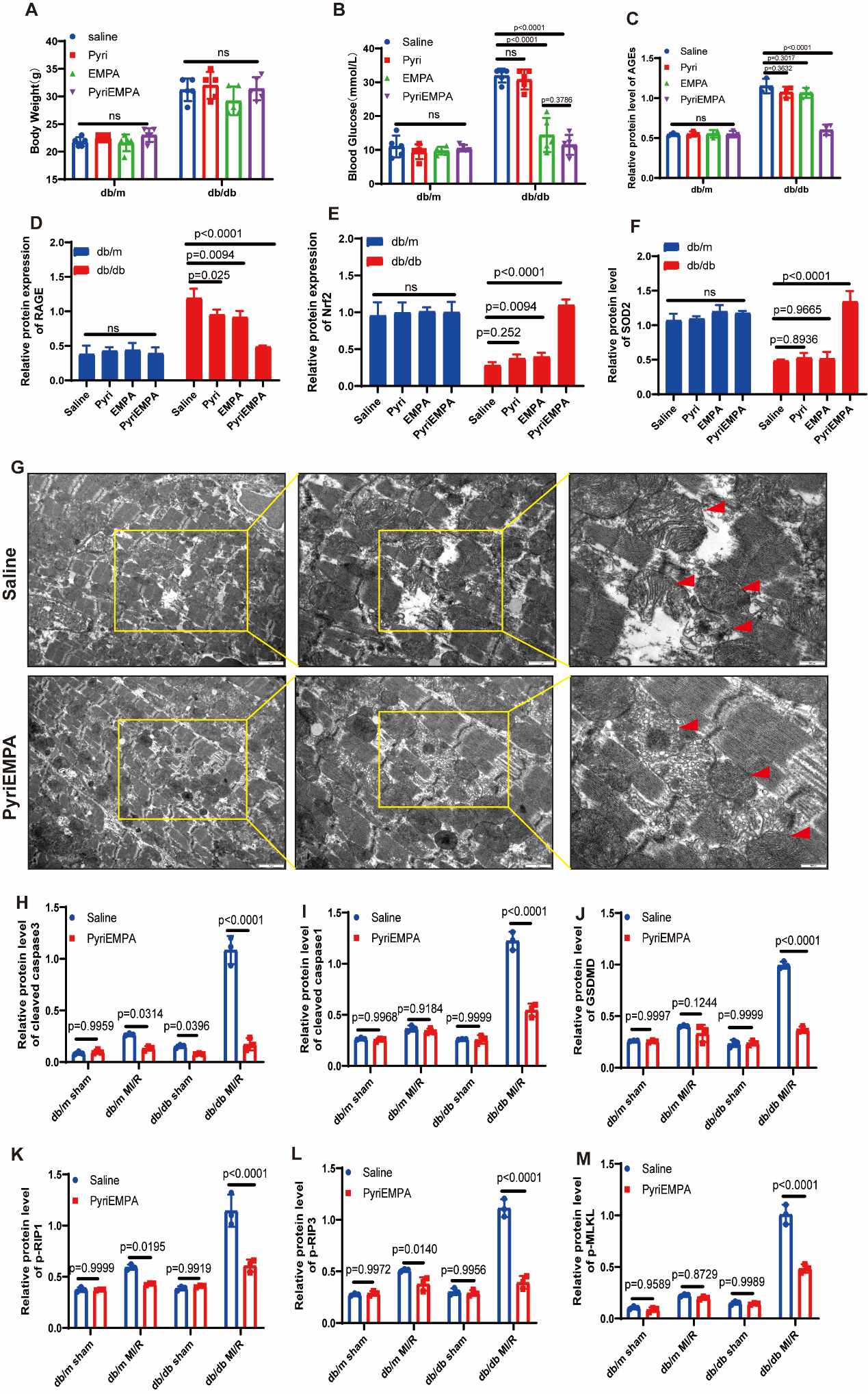


​​Body weight and **(B)** blood glucose of db/db (diabetic) and db/m (non-diabetic) mice receiving different treatments before MI/R surgery(n=5). (C) Serum AGEs concentration in the indicated groups, n (biological replicates) =5. Quantitative analysis of relative protein expression levels of **​​(D)​​**RAGE, **​​(E)**​​ Nrf2, **​​(F)**​​ SOD2 in heart tissues.**​​(G)​​** Representative TEM images of myocardial ultrastructure in non-diabetic mice treated with saline or PyriEMPA. Scale bar: 2μm/1μm/500nm. Quantitative analysis of relative protein expression levels of **​​(H)​​** cleaved caspase-3, **​​(I)**​​ cleaved caspase-1, **​​(J)**​​ GSDMD, **​​(K)​​** p-RIP1, **​​(L)**​​ p-RIP3, and **​​(M)**​​ p-MLKL in heart tissues. (A-F, H-M) *P*-value was calculated using one-way ANOVA followed by Tukey’s multiple comparisons test. Data were presented as mean ± SD. A two-tailed *P* < 0.05 was considered statistically significant.

**Supplementary Fig. 6**


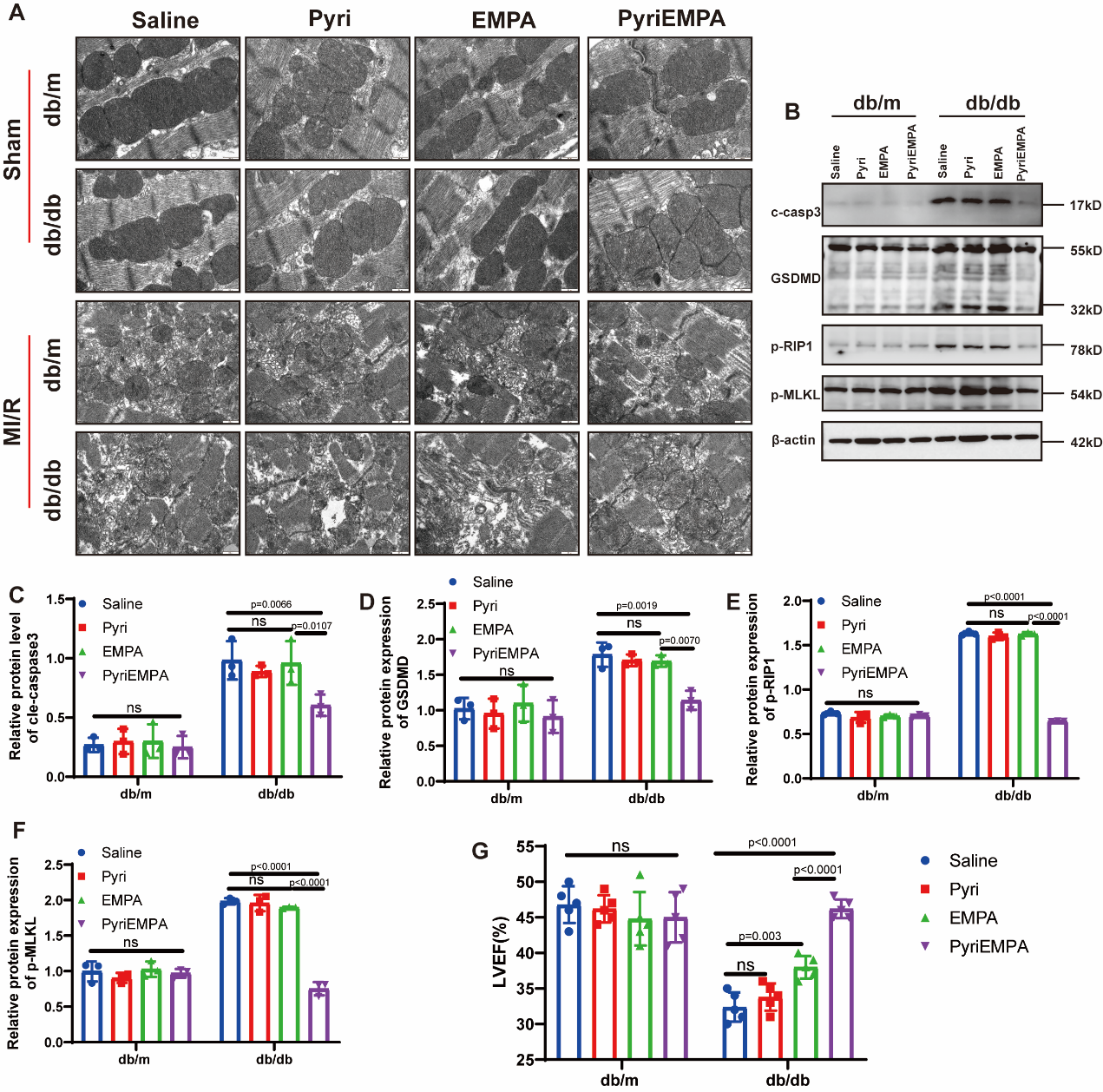


**(A)** Representative TEM images of myocardial ultrastructure in diabetic mice and non-diabetic mice treated with Saline, Pyridoxamine, empagliflozin, and PyriEMPA. Scale bar: 500nm. **(B)** Western blot analysis of PANoptosis executors. Quantitative analysis of relative protein expression levels of **​​(C)​​** cleaved caspase-3, **​​(D)**​​ GSDMD, **​​(E)​​** p-RIP1, **​​(F)**​​ p-MLKL in heart tissues. n (biological replicates) =3. ​**​(G)​**​ Left ventricular ejection fraction (LVEF) measured by echocardiography. n (the number of animals used) = 5 in each group. Data were presented as mean ± SD. (C-G) *P*-value was calculated using one-way ANOVA followed by Tukey’s multiple comparisons test. A two-tailed *P* < 0.05 was considered statistically significant.

**Supplementary table 1. Antibodies used in the present study.**

| Primary antibodies | | | |
| --- | --- | --- | --- |
| Target antigen | Catlog# | Vendor or Source | Working Solution |
| AGEs | ab23722 | Abcam | WB 1:1000  IHC 1:2000 |
| AIM2 | 63660 | CST | WB 1:1000  IHC 1:100 |
| ASC | 83858-3-RR | Proteintech | IHC 1:500 |
| Cleaved-caspase 3 | 9661 | CST | WB 1:1000 |
|  | AFRP0036 | AiFang Biological | IHC 1:300 |
| Cleaved-caspase 1 | 89332 | CST | WB 1:1000 |
| Cytochrome c | 4272 | CST | WB 1:1000 |
| GSDMD | ab209845 | Abcam | WB 1:1000 |
|  | ab219800 | Abcam | IHC 1:1000 |
| p-RIP1 | 53286 | CST | WB 1:1000 |
| p-RIP3 | 91702 | CST | WB 1:1000 |
| p-MLKL | 37333 | CST | WB 1:1000  IHC 1:1600 |
| NLRP3 | 15101 | CST | WB 1:1000 |
| Nrf2 | 20733 | CST | WB 1:1000 |
| RAGE | Ab216329 | Abcam | WB 1:1000 |
| SOD2 | 13141 | CST | WB 1:1000 |
| ZBP1 | AG-20B-0010-C100 | Adipogen | IHC 1:500 |
|  | 60968 | CST | WB 1:1000 |
| Secondary antibodies | | | |
| Goat Anti-Rabbit IgG H&L (HRP) | ab205718 | Abcam | 1:4000 |
| oat Anti-Mouse IgG H&L (HRP) | ab205719 | Abcam | 1:4000 |

**Supplementary Table 2. Primers and siRNAs used in the present study.**

| Primers | | |
| --- | --- | --- |
| Name | Sequence (5'-3') | |
| mtCOI | Forward：GCCCCCGATATGGCGTTT | |
|  | Reverse: TAAACTTCAGGGTGACCAAAAAATCA | |
| 18S | Forward：GCAATTATTCCCCATGAACG | |
|  | Reverse: GGCCTCACTAAACCATCCAA | |
| siRNA | | |
| Name | Sequence (5'-3') | LOT.NO. |
| si*Aim2* | sense: CAGAGAAGAUUUACAGCAA (dT)(dT) | NS-143276-021 |
|  | antisense: UUGCUGUAAAUCUUCUCUG (dT)(dT) | NS-143276-022 |
| si*RAGE* | sense: GCCGGAAAUUGUGAAUCCU (dT)(dT) | NS-143276-007 |
|  | antisense: AGGAUUCACAAUUUCCGGC (dT)(dT) | NS-143276-008 |
| si*Nrf2* | sense: GCACGGUGGAGUUCAAUGA (dT)(dT) | NS-143276-003 |
|  | antisense: UCAUUGAACUCCACCGUGC (dT)(dT) | NS-143276-004 |
| si*Zbp1* | sense: CACAGAUGUGGACCAUCUA (dT)(dT) | NS-143276-027 |
|  | antisense: UAGAUGGUCCACAUCUGUG (dT)(dT) | NS-143276-028 |
| Primers and siRNAs used in the present study are synthesized by Beijing Tsingke Biotech Co., Ltd. | | |
